# Polymorph Impact on the Bioavailability and Stability of Poorly Soluble Drugs

**DOI:** 10.3390/molecules201018759

**Published:** 2015-10-15

**Authors:** Roberta Censi, Piera Di Martino

**Affiliations:** School of Pharmacy, University of Camerino, via S. Agostino, 1, Camerino 62032, Italy; E-Mail: roberta.censi@unicam.it

**Keywords:** polymorphism, poorly soluble drug, polymorphism screening, regulatory issues

## Abstract

Drugs with low water solubility are predisposed to poor and variable oral bioavailability and, therefore, to variability in clinical response, that might be overcome through an appropriate formulation of the drug. Polymorphs (anhydrous and solvate/hydrate forms) may resolve these bioavailability problems, but they can be a challenge to ensure physicochemical stability for the entire shelf life of the drug product. Since clinical failures of polymorph drugs have not been uncommon, and some of them have been entirely unexpected, the Food and Drug Administration (FDA) and the International Conference on Harmonization (ICH) has required preliminary and exhaustive screening studies to identify and characterize all the polymorph crystal forms for each drug. In the past, the polymorphism of many drugs was detected fortuitously or through manual time consuming methods; today, drug crystal engineering, in particular, combinatorial chemistry and high-throughput screening, makes it possible to easily and exhaustively identify stable polymorphic and/or hydrate/dehydrate forms of poorly soluble drugs, in order to overcome bioavailability related problems or clinical failures. This review describes the concepts involved, provides examples of drugs characterized by poor solubility for which polymorphism has proven important, outlines the state-of-the-art technologies and discusses the pertinent regulations.

## 1. Introduction

In the industrial development of a new drug substance and/or product, considerable problems are posed by candidate drugs with poor aqueous solubility, as this characteristic is related to poor bioavailability. Research and Development takes various approaches to enhancing the solubility and/or dissolution rate, and thus oral bioavailability, of poorly water-soluble drugs. One of the most common and effective approaches for increasing the solubility and dissolution rates of acidic and basic drugs is salt formation [1]. More recently, co-crystals, defined as crystalline materials comprised of at least two different components [2], have attracted attention for improving the dissolution rate of poorly water-soluble drugs [3]. Drug particle size reduction, affecting the dissolution rates, has been revealed one of oldest strategies for improving bioavailability of drugs and has been frequently applied in the pharmaceutical industry for routine production [4]. During the last years, the development of nanotechnologies have aroused the interest of researchers who have developed new technologies, easily industrially scalable, to reduce the particle size to nanodimensions [5,6]. Including or dispersing the poorly soluble drug in a carrier such as a cyclodextrin [7,8] or a polymer (solid dispersion) [9] are also common applied approaches. Modifications in the solid state, conversion from one polymorph to another [10], solvation/hydration [11], or amorphization [12,13] have been intentionally or unintentionally considered by the researchers and by the pharmaceutical industry during drug development of poorly-soluble drugs.

When the polymorphic form modification approach is chosen, not only must the effective improvement of drug bioavailability—which is not always obvious—be verified, but problems with the drug substance and product stability can arise. Generally, metastable forms are more soluble than the corresponding stable polymorphic forms, but they transform to the more thermodynamically stable form in a relatively short time [14], and thus it is necessary to monitor the polymorphic transformation during formulation, manufacturing, and storage of dosage forms to ensure reproducible bioavailability after administration [15].

In addition, the change of the polymorphic form has frequently caused clinical failures once it is on the market. This review should provide a useful overview for pharmaceutical industry readers interested in the development of new drug substances and/or products using polymorphic modifications, and offers many examples of such efforts.

Since the US Food and Drug Administration (FDA) and the International Conference on Harmonization (ICH) classify anhydrous, hydrate and solvate forms as polymorphs [16], in this review the term polymorphism will refer to both anhydrous and solvate (hydrate) forms.

## 2. Importance of Solubility on the Bioavailability of Drugs

Solubility is the ability of a *solute* to dissolve in a *solvent* to form a homogeneous solution of the solute in the solvent. This property is influenced by temperature and pressure [17]. Typical aqueous solubilities are indicated in several Pharmacopoeia, including the U.S. Pharmacopoeia (Table 1).

Solubility is an essential property of drugs, because they must dissolve in order to be absorbed through membranes and reach the site of action. Consequently, solubility is one of the most critical and important parameters influencing drug bioavailability, that is, the ability of a drug to be available in an appropriate concentration at the site of action, independently of the pharmaceutical dosage form and route of administration.

**Table 1:** Aqueous solubilities according to the U.S. Pharmacopoeia.

Freely Soluble	100–1000 mg/mL
Soluble	33–100 mg/mL
Sparingly soluble	10–33 mg/mL
Slightly soluble	1–10 mg/mL
Very slightly soluble	0.1–1 mg/mL
Practically insoluble	<0.1 mg/mL

Poor aqueous solubility is the one of the major problems encountered during the development of new drug substances and/or drug products. This aspect becomes even more important if one considers that more than 40% of marketed immediate release oral drugs are practically insoluble (<100 g/mL) [18,19], and that fully 70% of new drug candidates in the pharmaceutical industry pipelines are practically insoluble in water [20]. Jean-Paul Garnier, CEO of GlaxoSmithKline, said that “*About 50% of drug candidates that enter clinical trials fail due to efficacy and safety concerns, and the remaining 40% fizzle due to patent concerns and issues like solubility and drug interaction*” [21].

To have an idea of the importance of drug solubility and how poor aqueous solubility limits drug bioavailability, one can refer to some examples offered by Amidon *et al.*, [22] concerning the volumes needed to dissolve poorly water soluble drugs according to their dose. Some of the consequences of the inadequate aqueous solubility of a drug are limited and variable absorption, formulation and food effects, and poor tissue distribution and metabolism [23].

The importance of the solubility parameter is confirmed in the Biopharmaceutical Classification System (BCS) [24], a scientific framework for classifying drug substances based on their aqueous solubility and intestinal permeability [22,25]. The BCS takes into account three major factors that govern the rate and extent of drug absorption from immediate release solid oral dosage forms: (1) dissolution; (2) solubility; and (3) intestinal permeability. According to the BCS, drug substances are classified as follows:

Class 1: High Solubility–High Permeability
Class 2: Low Solubility–High Permeability
Class 3: High Solubility–Low Permeability
Class 4: Low Solubility–Low Permeability

## 3. The Polymorphism of Drugs: Anhydrous and Solvated Forms

Among the various techniques used to enhance the solubility of poorly soluble drugs are physical and chemical modifications of the drug, and methods such as particle size reduction, salt formation, solid dispersion, use of surfactant, and complexation [23]. Selection of a solubility improving method depends on drug property, site of absorption, and required dosage form characteristics [26].

Crystalline polymorphs have the same chemical composition, but different internal crystal structures, and therefore possess different physicochemical properties [27] because of their different lattice structures and/or different molecular conformations [28]. The phenomenon of polymorphism is quite common among organic molecules, and many drugs can crystallize into different polymorphic forms [29,30,31,32].

Polymorphic forms of drugs can prove interesting for drug developers because their thermodynamic and physicochemical properties, such as energy, melting point, density, stability, and in particular solubility, may offer an improvement on the original form.

Generally, the solubility of metastable polymorphs is kinetically higher than that of a thermodynamically more stable polymorph [33], offering, at least in theory, a solution to bioavailability problems.

Actually, it has been demonstrated that differences between the solubility of one polymorph and another are typically lower than a factor of 2 [34] or more rarely of 5 [35]. Thus, while a polymorph may offer a slight improvement in solubility compared to the original compound, this benefit may be offset the fact that it is also less stable than the original, and thus there may be no advantage in choosing this polymorph over the original compound. Actually, metastable and more soluble forms tend to convert into the more thermodynamic stable form in a relatively short time. The presence of specific excipients, or particular chemical and/or technological processes may accelerate the transition to the solid state [36,37]. This transition may proceed according to the relative thermodynamic stability of metastable forms, or be accelerated by the presence of seeds of one polymorph in another, with important repercussions on clinical practice, as it was the case of ritonavir (refer to the specific paragraph).

Solvates, also inappropriately termed pseudopolymorphs [38], are crystalline solids containing within the crystal structure either stoichiometric or nonstoichiometric proportions of solvent. When the incorporated solvent is water, the solvate is called a hydrate [27]. In general, it is undesirable to use solvates for drugs and pharmaceuticals, as the presence of organic solvent residues may be toxic; regulations for all the organic solvents in products for human use establish specific limits to how much daily exposure to residual solvent in the formulated preparation is allowed.

The solubility and dissolution rate of a drug can significantly differ for different solvates, and in particular hydrates. Important reviews concerning pharmaceutical solvates and hydrates are those of Morris [39] and Khankari and Grant [11].

Hydrates may have a faster or slower dissolution rate than the corresponding anhydrous form, though more frequently, the former are slower than the latter [40], perhaps because there are fewer sites of the drug molecule available for interaction with water during dissolution. A classic example is theophylline anhydrate, which dissolves faster than its hydrate form [41,42].

In other cases, the hydrate form exhibits a more rapid dissolution rate than its anhydrous form: for example, erythromycin dihydrate was found to exhibit a significantly faster dissolution rate than that of monohydrate and anhydrous forms [43,44].

Glibenclamide has been isolated as pentanol and toluene solvates, and these solvates exhibited higher solubility and dissolution rate than two non-solvated polymorphs [45].

The physical stability of hydrates and anhydrous forms strongly depends upon the relative humidity and/or temperature of the environment [46,47,48], and transitions from one form to the other occur as a consequence of variations in storage conditions and/or technological treatments [37,49].

In particular, anhydrous to hydrate transitions can occur during dissolution at the drug/medium interface and can affect dissolution rate and perhaps bioavailability [46].

## 4. Polymorph Screening

The polymorph screening process seeks to determine whether a given compound exists in polymorphic forms [50]. In recent decades, several techniques have been developed to improve the polymorph screening of drugs.

The concept of crystal engineering was introduced by Pepinsky in 1955 [51] and first applied by Schmidt in the context of covalent bond formation in the solid state [52]. It is traditionally defined as the deliberate design and control of molecular packing within a crystal structure with the intention of generating a solid that shows a particular desirable characteristic [53,54,55]. Combinatorial chemistry and high-throughput screening used in drug discovery have resulted in an increase of poorly water soluble drug candidates [56,57].

Among traditional methods to generate polymorphs (as well as hydrates and solvates), manual techniques [58] are time and material consuming, and sometimes fail to identify all possible polymorphs for a compound.

The development of computer software tools that consider the arrangement of atoms within a compound to predict the possible crystal structures has been a boon to the pharmaceutical industryenabling savings of time and materials in the process of identifying the most thermodynamically stable polymorph, and making it possible to tailor the manufacturing process for production of the active ingredient [59].

High-throughput polymorphism screening has been developed with the aim of accelerating the identification of potential polymorphs for a drug, and thus avoid problems during drug development [60,61]. The efficiency of screening in HT mode is estimated to be about two orders of magnitude greater than that of traditional bench-scale approaches [62], and it has been applied to numerous drugs.

A high-throughput (HT) crystallization study of an experimental angiotensin II antagonist and sertraline hydrochloride identified new forms, improved understanding of the transitions among different forms, and demonstrated that an HT strategy coupled with critical analysis can be used to rank the usefulness of crystal forms [62].

Ritonavir is a drug that has been used to treat HIV-1 infections since 1996. In 1998, a new metastable and unknown form posed major bioavailability problems. Afterwards, HT screening identified a total of five forms, the two well-known forms and three unknown ones [60].

A high-throughput co-crystal slurry screening study of indomethacin that used *in situ* Raman microscope and a multi-well plate not only provided information about co-crystal formation within one day, but also yielded data about the equilibrium of co-crystal formation and polymorphic transformation in just one screening [63].

## 5. Case Studies of Polymorphic Drugs

The following paragraphs report several examples of poorly soluble drugs for which polymorphic issues proved important. A summary is given in Table 2.

**Table 2:** Summarization of polymorphism of several drugs.

Drug Substance	Polymorphism Aspects	Bioavailability Issues
Chloramphenicol palmitate 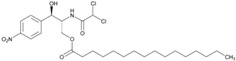	Chloramphenicol palmitate is a prodrug of chloramphenicol with antibiotic properties [64]. Chloramphenicol palmitate exist in three polymorphic forms: (A, B, C) [65,66], the stable form A (biologically inactive modification), the metastable form B (active modification) and unstable form C [67,68,69]. The three crystalline forms were also called α, β and γ. The α form is unstable at room temperature and gradually transforms to β on storage [70,71].	Form B (β) dissolves faster than Form A (α), and has a much higher solubility [72,73,74]. Low serum levels for the stable polymorph A were observed [75].
Oxytetracycline 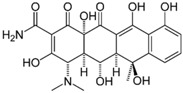	Oxytetracycline is a broad spectrum antibiotic. It exists in two different polymorphs [76].	Oxytetracycline showed differences in patients’ blood levels [77] or differences in *in vitro* dissolution of tablets [78] because of differences in polymorphic forms.
Carbamazepine 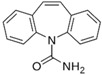	Carbamazepine is used in the treatment of epilepsy and trigeminal neuralgia. Different polymorphic forms were described [79,80,81,82,83,84,85,86,87,88,89,90,91]. Four anhydrous polymorphs were characterized: I, II, III, and IV, respectively identified as triclinic, trigonal, monoclinic, and monoclinic [77].	In spite different studies demonstrated similar pharmacokinetics in humans of anhydrous and dihydrate forms of carbamazepine [92] and no differences in bioavailability between a generic carbamazepine product and an innovator product [93], several clinical failures were reported concerning carbamazepine [94,95], in particular with generic carbamazepine tablets [96]. More recently, it was confirmed that the initial dissolution rate of carbamazepine was in the order of form III > form I > dihydrate, while the order of AUC values was form I > form III > dihydrate. This discrepancy may be attributed to the rapid transformation from form III to dihydrate in GI fluids [97].
Ritonavir 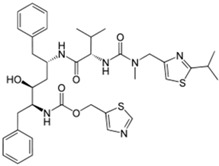	Ritonavir is an antiretroviral drug belonging to protease inhibitor class and used to treat HIV-1 infection. Ritonavir exhibits conformational polymorphism [98] and a total of five forms were described [60]. The forms I and II were more extensively characterized [98].	2 years after the launch of the first ritonavir product, several batches failed dissolution specifications because the presence of a different polymorphic form having ~50% lower intrinsic solubility of reference form [36].
Atorvastatin calcium 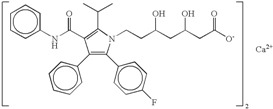	Atorvastatin calcium is an inhibitor of 3-hydroxy-3-methylglutaryl-coenzyme A (HMG-CoA) reductase, with strong ability to lowering blood cholesterol. At least 60 polymorphic forms/solvates/hydrates have been patented [99,100,101]. It is not unusual to verify the presence of polymorphic impurities in the marketed atorvastatin calcium (API) with consequences on drug bioavailability and stability [102].	Atorvastatin is unstable and the hydroxy acid form is converted to lactone form that is 15 times less soluble than the hydroxyl acid form [103,104]. This instability of atorvastatin calcium leading to poor solubility (0.1 mg/mL) is the main cause for low bioavailability of the drug after oral administration as the absolute bioavailability of atorvastatin calcium is only 14% [105].
Axitinib 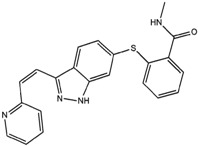	Axitinib is a tyrosine kinase inhibitor of endothelial growth factor interrupting tumor angiogenesis and thus, preventing the growth of cancer cells. 60 solvates, polymorphs of solvates, and five anhydrous forms were discovered [106,107,108,109].	The commercial formulation under trade name Inlyta^®^ contains the stable anhydrous form [107].
Phanylbutazone 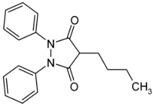	Phenylbutazone is a potent anti-rheumatic drug existing in different polymorphic and solvated forms [110,111,112,113]. Anhydrous forms I and II were more extensively described and form II resulted more soluble than form I. The Form III is a highly unstable form [110].	Anhydrous forms I and II polymorphic forms exhibited different solubilities, dissolution rates and oral absorption [110,114].
Rifaximin 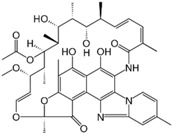	Rifaximin is a synthetic derivative of rifamycin, with very low gastrointestinal absorption, but still displaying a broad spectrum of antibacterial activity [115,116,117]. Rifaximin shows crystal polymorphism (poolymorphs α, β, γ, δ, ε) [118,119]. The polymorph α is the most thermodynamically stable form and the commercial one.	In vitro studies show different dissolution and solubility rates for these polymorphs, and *in vivo* investigations in dogs found different pharmacokinetic patterns, with δ and γ polymorphs displaying the highest systemic bioavailability [119]. The most PK parameters were significantly higher after administration of generic rifaximin, because of the presence of both rifaximin-α and amorphous forms [120].

### 5.1. Chloramphenicol Palmitate

A decades-old classic example of the importance of polymorphism to bioavailability is chloramphenicol palmitate, a prodrug of chloramphenicol with antibiotic properties, developed with the objective of obtaining a more pleasant flavored derivative [64]. Chloramphenicol palmitate exists in three polymorphic forms [65,66,70,71], the stable form A (biologically inactive modification), the metastable form B (active modification) and the unstable form C [67], which recently have been fully characterized thanks to advances in analytical methods [68,69]. Polymorph A is the thermodynamically stable one, but its absorption in humans is significantly lower than that of polymorph B [72], because Form B dissolves faster than Form A, and has much higher solubility [73]. This solubility difference probably results in the difference in ester hydrolysis rates, and thus in the difference in oral absorption, if one considers that chloramphenicol palmitate must be hydrolyzed by intestinal esterases before it can be absorbed [74]. These results were also proven by the low serum levels reached by the stable polymorph A, whereas the metastable polymorph yielded much higher serum levels when the same dose was administered [75].

### 5.2. Oxytetracycline

While for many years it has been known from various studies that patient blood levels of oxytetracycline differed according to the supplier of the oxytetracycline capsules, [77] or that *in vitro* dissolution performance of oxytetracycline tablets differed according to the various sources [78], only more recently have these differences been attributed to the presence of different polymorphs [76]. Tablets prepared from the form A polymorph dissolved significantly more slowly than tablets prepared from polymorph B: indeed, the tablets with form *A* polymorph exhibited about 55% dissolution at 30 min, while the tablets with form *B* polymorph exhibited almost complete (95%) dissolution at the same time. Further studies characterizing the physical and chemical properties of oxytetracycline polymorphs would be useful, as no recent works are available in the literature.

### 5.3. Carbamazepine

Highly different polymorphic forms of carbamazepine, a drug used in the treatment of epilepsy and trigeminal neuralgia, were discovered through classical crystallization methods and fully characterized from a physicochemical point of view [79,80,81,82,83,84,85,86,87,88,89]. More recently, a crystal engineering design strategy has facilitated supramolecular synthesis of 13 new crystalline phases of carbamazepine [90].

Even though different studies demonstrated that anhydrous and dihydrate forms of carbamazepine have similar pharmacokinetics in humans [92], and another indicated that there are no differences in bioavailability between a generic carbamazepine product and an innovator product [93], several clinical failures with carbamazepine were reported [94,95]. In particular, several problems were observed with Generic carbamazepine tablets, which were recalled due to clinical failures and dissolution changes [96]. It was suggested that discrepancies in clinical parameters and irreproducible clinical behavior within different batches and suppliers of the generic carbamazepine tablets were due to moisture uptake during storage. Actually, it is well known that anhydrous carbamazepine converts to the dihydrate within 1 h, when the anhydrous form is suspended in water [91]. More recently, it was confirmed that the initial dissolution rate of carbamazepine was in the order of form III > form I > dihydrate, while the order of AUC values was form I > form III > dihydrate. This discrepancy may be attributed to the rapid transformation from form III to dihydrate in GI fluids [97].

### 5.4. Ritonavir

Ritonavir, an antiretroviral drug of the protease inhibitor class used to treat HIV-1 infections, was found to have polymorphism that strongly impacts on solubility and dissolution rate. Originally, only one form was described, and was formulated as soft gel capsules containing an ethanol/water solution molecule. Two years after the launch of the product, several batches failed dissolution specifications. A new thermodynamically stable Form II was discovered, but this form precipitated out of solution, having ~50% lower intrinsic solubility than the reference form. This finally forced the manufacturer to recall the original formulation from the market [36] and reformulate it in an oily vehicle.

Using solid state spectroscopy and microscopy techniques including solid state NMR, Near Infrared Spectroscopy, powder X-ray Diffraction and Single crystal X-ray, ritonavir was found to exhibit conformational polymorphism with two unique crystal lattices that have significantly different solubility properties [98]. In addition, HT screening identified a total of five forms, the two well know forms and three unknown ones [60].

### 5.5. Atorvastatin Calcium

Atorvastatin calcium is an inhibitor of 3-hydroxy-3-methylglutaryl-coenzyme A (HMG-CoA) reductase, with strong ability to lower blood cholesterol. Atorvastatin, the most preferred molecule among statins, was developed and marketed by Pfizer under the trade name Lipitor^®^ [121] and was the number one selling drug in the US until its patent expired in 2011. Atorvastatin is unstable and the hydroxyacid form (HF) is converted to a lactone form (LF), which is 15 times less soluble than the hydroxyacid form [103,104]. This instability of atorvastatin calcium leading to poor solubility (0.1 mg/mL) is the main cause for low bioavailability of the drug after oral administration: the absolute bioavailability of ATC is only 14% [105]. 

At least 60 polymorphic forms/solvates/hydrates have been patented [99,100,101] and several pharmaceutical companies are developing or have developed generic drug formulations based on different atorvastatin calcium polymorphs.

Due to the patent expiration, several companies produce the active pharmaceutical ingredient (API) of atorvastatin calcium, available on the market as stable crystalline polymorph I or amorphous form. It was not unusual to verify the presence of polymorphic impurities in the marketed atorvastatin calcium (API) with consequences on drug bioavailability and stability [102].

### 5.6. Axitinib

Axitinib is a tyrosine kinase inhibitor of endothelial growth factor that interrupts tumor angiogenesis and thus prevents the growth of cancer cells. Because of its strong molecular flexibility, 60 solvates, polymorphs of solvates, and five anhydrous forms have been discovered [106,107,108,109]. The commercial formulation under trade name Inlyta^®^ contains the stable anhydrous form. Unusually, conventional crystallization methods did not lead to the discovery of this most stable polymorph; rather, it was obtained by the uncommon method of slurrying the solvates at high temperature. Understanding of the desolvation pathway was critical for obtaining the most stable polymorph of axitinib [107].

### 5.7. Phenylbutazone

Phenylbutazone is a potent anti-rheumatic drug that exists in different polymorphic [110,111,112] and solvated forms [113]. Different solubilities, dissolution rates and oral absorption were highlighted between two different polymorphic forms [114].

### 5.8. Rifaximin

Rifaximin is a synthetic derivative of rifamycin with very low gastrointestinal absorption, but that nonetheless displays a broad spectrum of antibacterial activity [115,116,117]. According to the European Pharmacopoeia, rifaximin shows crystal polymorphism [118] and several polymorphs (α, β, γ, δ, ε) have been described [119]. The most thermodynamically stable form, polymorph α, is the one used commercially. *In vitro* studies show different dissolution and solubility rates for these polymorphs, and *in vivo* investigations in dogs found different pharmacokinetic patterns, with δ and γ polymorphs displaying the highest systemic bioavailability [119]. Blandizzi *et al.*, [120] compared one generic rifaximin formulation with the branded product (the latter containing only polymorph-α) and found that most PK parameters such as highest concentration achieved in plasma (C_max_), area under the concentration-time curve (AUC), and cumulative urinary excretion were significantly higher after administration of generic rifaximin. X-ray power diffraction analysis of the generic formulation showed the presence of both rifaximin-α and amorphous rifaximin, which could have contributed to the increased systemic bioavailability of the generic formulation.

## 6. Regulatory Considerations

For approval of a new drug, the drug substance guideline of the US Food and Drug Administration (FDA) states that “appropriate” analytical procedures need to be used to detect polymorphs, hydrates and amorphous forms of the drug substance and also stresses the importance of controlling the crystal form of the drug substance during the various stages of product development [122].

Modern techniques such as ss-NMR and NIR can identify polymorphs in dosage forms (within limits), and should help improve mechanistic understanding of polymorphs in future studies [123]. Fast and easily applicable techniques such as DSC can determine the solubility of different polymorphs very rapidly and accurately [124]. The selection of crystal forms of improved solubility and bioavailability is possible when appropriate strategies are applied to guarantee the drug stability over the shelf life of the drug product. The evaluation of crystal transitions through appropriate analytical technologies serves to predict unwanted conversions during the drug product shelf life.

## 7. Conclusions

The possibility of detecting drug polymorphism can be viewed in two opposite ways: as a risk of clinical failure when an undesired solid state conversion occurs, or as an advantage when more soluble polymorphs may be selected to overcome bioavailability problems. Thus, the pharmaceutical industry must carefully evaluate the presence of the phenomenon of the polymorphism for every drugs under development. In the past, when analytical techniques were not sophisticated enough to adequately detect polymorphism of drugs under development, several clinical failures emerged during the marketing phases, in some cases with serious repercussions for the pharmaceutical industry, such as the obligation to withdraw or reformulate the product. Now, the use of state-of-the-art technologies makes it possible to prevent this risk and to better and fully investigate the existence of different polymorphic forms of drugs in the industrial pipeline. In recent years, regulatory organisms such as the FDA and ICH have pressed the pharmaceutical industry to adopt methodologies and innovative analytical techniques that should provide better understanding of the polymorphism phenomenon for every drug under development, and enable Quality Control Departments to adequately evaluate the solid state of batches produced.
